# Unusual oxidation-induced core-level shifts at the HfO_2_/InP interface

**DOI:** 10.1038/s41598-018-37518-2

**Published:** 2019-02-06

**Authors:** Jaakko Mäkelä, Antti Lahti, Marjukka Tuominen, Muhammad Yasir, Mikhail Kuzmin, Pekka Laukkanen, Kalevi Kokko, Marko P. J. Punkkinen, Hong Dong, Barry Brennan, Robert M. Wallace

**Affiliations:** 1 Department of Physics and Astronomy, University of Turku, FI-20014 Turku, Finland; 2 Ioffe Physical-Technical Institute, Russian Academy of Sciences, St. Petersburg, 194021 Russian Federation; 3 Department of Materials Science and Engineering, The University of Texas at Dallas, Richardson, Texas 75080 USA; 4 Present Address: Department of Electronics and Tianjin Key Laboratory of Photo-Electronic Thin Film Device and Technology, Nankai University, Tianjin, 300071 China; 5 Present Address: National Physical Laboratory, Hampton Road, Teddington, TW11 0LW United Kingdom

## Abstract

X-ray photoelectron spectroscopy (XPS) is one of the most used methods in a diverse field of materials science and engineering. The elemental core-level binding energies (BE) and core-level shifts (CLS) are determined and interpreted in the XPS. Oxidation is commonly considered to increase the BE of the core electrons of metal and semiconductor elements (*i*.*e*., positive BE shift due to O bonds), because valence electron charge density moves toward electronegative O atoms in the intuitive charge-transfer model. Here we demonstrate that this BE hypothesis is not generally valid by presenting XPS spectra and a consistent model of atomic processes occurring at HfO_2_/InP interface including negative In CLSs. It is shown theoretically for abrupt HfO_2_/InP model structures that there is no correlation between the In CLSs and the number of oxygen neighbors. However, the P CLSs can be estimated using the number of close O neighbors. First native oxide model interfaces for III-V semiconductors are introduced. The results obtained from *ab initio* calculations and synchrotron XPS measurements emphasize the importance of complementary analyses in various academic and industrial investigations where CLSs are at the heart of advancing knowledge.

## Introduction

The x-ray photoelectron spectroscopy (XPS) is widely utilized not only in the characterization of the chemical composition of materials but also to understand and control various scientifically and industrially interesting phenomena such as atomic layer deposition, catalysis, materials protection, operation of electronic devices, and photoelectrochemical reaction (*e*.*g*., refs^1–15^). In research and development of these phenomena, the main XPS objective is typically determination and interpretation of the CLS, which are further combined with results of other measurements to obtain interrelationships between important properties. The CLSs are commonly interpreted in terms of electronegativity differences between elements. Excess (deficit) charge in the valence shell of an atom decreases (increases) the BE of a core electron according to the classical electrostatic case of the potential inside a uniformly charged spherical surface. Charge transfer in oxides is often expressed in terms of the oxidation state. This interpretation applies nicely to silicon oxidation, because a Si atom has four valence electrons. Therefore, the oxidation number of a silicon atom (0, +1, +2, +3, +4) is equal to the number of oxygen neighbors. Coincidentally, even the numerical values of the CLSs of the Si atoms (in eVs) equal roughly to the oxidation numbers^6^. The CLSs of other oxides are interpreted often in the same way. The BE is increased with the number of oxygen neighbors. However, it is much less clear, to what extent this model can be applied to other, especially more complex systems like oxide/III-V semiconductor interfaces. In general, the CLSs depend on several factors, not just on the atomic on-site charge and different complex environments can induce similar CLSs.

In this work, we report that the semiconductor oxidation can surprisingly cause negative CLSs by presenting theoretical and experimental results for the HfO_2_/InP junction. In more general terms, the presented results reveal how one should be cautious when analyzing the XPS spectra solely in terms of the electronegativities of elements and number of oxygen neighbors. Furthermore, the oxidation-induced CLSs of a semiconductor are interpreted, which is further essential to understand phenomena like the ALD mechanisms^4^ and the formation of surface defects harmful to electronics and photonics devices^10,16–19^. The HfO_2_/InP interface is a prototypical insulator/semiconductor junction and also a potential component for devices like transistors^10,16–24^, nanowire solar cells^25–27^, and infrared detectors^28,29^. In these applications, HfO_2_ on InP typically acts as a dielectric and/or passivates the semiconductor crystal against environment-induced changes. It is essential to minimize the amount of interfacial defects, which can cause for example the Fermi-level pinning, non-radiative recombination, and leakage currents via the defect-induced electron states. Significant progress has been made in reducing the densities of such harmful band gap states (e.g., refs^10,16–24^). The development of atomic layer deposition (ALD) of insulator films has significantly aided this progress. Still, HfO_2_/III-V junctions contain too many defects as compared to the strict industrial reference of HfO_2_/Si. To reduce the defect concentration and to improve device performance, it is crucial to understand and control the chemical and physical properties of the HfO_2_/III-V interfaces, where III-V crystals become oxidized. XPS has been widely utilized in the studies to find interrelationships between the chemical composition and electrical properties of the HfO_2_/III-V interface^10,16–19^.

In this work, calculated CLSs based on the *ab initio* models for HfO_2_/InP have been combined with synchrotron-radiation XPS measurements of the HfO_2_/InP junctions grown by ALD. We focus on the CLSs of In *3d* and P *2p*, which are obtained with high enough resolution and surface sensitivity concerning the analysis made here, and yield well distinguishable changes as a function of photon energy and different sample treatments.

## Results

We first discuss the differences in the spectra as a function of probing depth and sample treatments, starting from P 2*p* that exhibits the most systematic differences, then move on to In 3*d* and see how In bonding is changed with respect to P. The interpretations made are also supported by supplementary information with Hf 4 *f* and S 2*p* spectra (see Fig. S1). These effects are then related to, and compiled consistently with the complementary data from computational results.

### P 2*p* measurements

**Figure 1:**
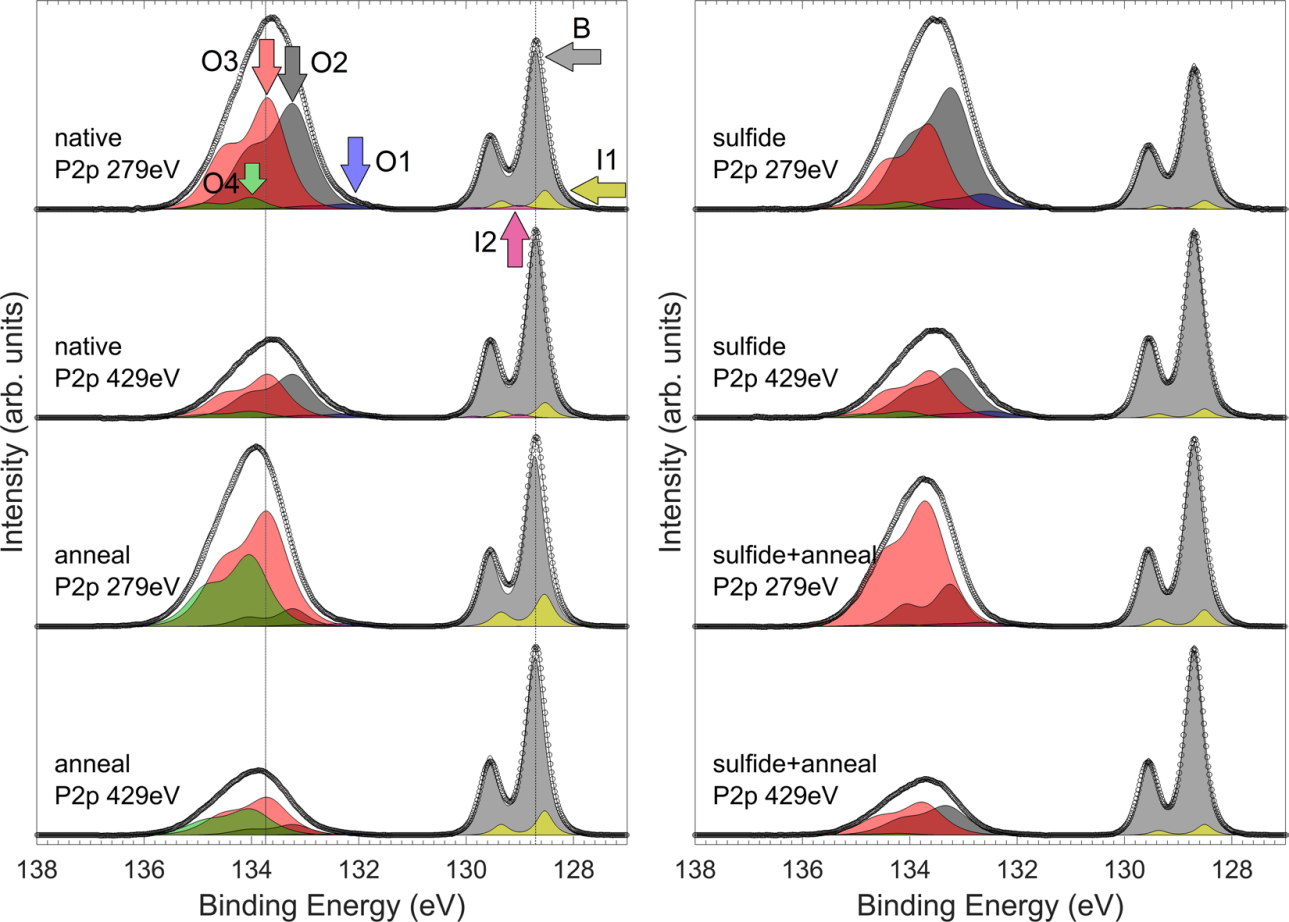
P *2p* spectra with fitted peaks. Vertical lines have been placed to illustrate the clear shift of the envelope of the O components even though B has been calibrated to 128.7 eV in these figures. On the left side the measurements of the corresponding native oxide experiments are shown, and sulfide treated on the right side. The energy label shows the chosen hν of the photons.

From Fig. 1 it can be seen that the P 2*p* emission around the InP bulk peak (about 129 eV, used as reference) is very narrow and exhibits the well-defined 2*p* doublet, in contrast to a broad oxide-related emission at 134 eV. The deconvolution of the P 2*p* spectra around the InP bulk emission is however complicated by the fact that the branching ratio varies from 0.4 to 0.47, instead of the theoretically predicted 0.5, when only single component (*i*.*e*., both 2*p*_3/2_ and 2*p*_1/2_ peaks) is included in the fitting of the emission at 129 eV. Thus additional components, I1 and I2, are introduced for consistency. It should be noted that particularly I2 is not very reliable due to only slight CLS which results in large changes in intensity ratios when the shifts are varied by even +/− 0.05 eV.

The other P 2*p* emission components: O1, O2, O3 and O4 are necessary to reproduce the characteristics of the emission around 134 eV. It has been commonly considered that native oxide of InP causes features at 134 eV due to P containing oxides at P^+5^ oxidation state, such as InPO_4_. Components at this BE have also been attributed to In(PO_3_)_3_, or could be related to InPHfO species. Here we have not observed P^0^ –type emission around +1 eV, which has been usually associated with pure P-P bonding or P clusters (*e*.*g*.^30,31^).

To suggest justified interpretations about the origin of each fitted component, we will discuss the depth distribution from which each component arises. The relative intensities of the components in the P 2*p* spectra have been listed in Table 1. Furthermore, to understand the relative depth from which the component arises, the peak intensities have been scaled with the bulk peak intensity of the corresponding measurement. Then the obtained values for the 150 eV kinetic energy (KE) measurement before and after annealing are divided by the corresponding values of the 300 eV KE measurement. This analysis provides the proportional increase of a component in the topmost layers; the higher value, the nearer to the outer surface a component arises. All of the obtained values for significant components other than B are >1, meaning that they are closer to the surface than bulk, as expected. I2 makes an exception, but its intensity is so low that reliable intensity analysis is not possible for I2. Furthermore, when the values for the annealed sample obtained this way are divided by the corresponding ones before the annealing, it can be deduced how much the average depth distribution of each signal changed due to the annealing treatment. If the average distribution would stay in place without movement of the average position, the last ratio would remain unity, since the distance from bulk would not change. This approach is especially sensitive to the changes in the topmost surface layers due to exponential dependence of the signal on emission depth. It is to be noted that, due to the exponential dependence, this ratio will remain unity, if there is slight broadening or movement of the specific state towards the surface, and simultaneously significant broadening or movement towards the bulk.

**Table 1:** First columns (under “Signal intensity”) give the proportional intensities of the fitted peaks for P *2p*_*3/2*_ for each measurement.

	Signal intensity (%)				Surface (150 eV) to bulk (300 eV) signal ratio (each peak referred to bulk peak intensity)		
	native 150 eV	native 300 eV	anneal 150 eV	anneal 300 eV	native	anneal	(anneal/native)
B (0 eV)	28,6	49,8	29,1	48,9	1,00	1,00	1,00
I1 (−0.18 eV)	1,9	2,0	5,2	6,7	1,66	1,30	0,79
I2 (+0.30 eV)	0,6	0,2	0,7	2,9	4,35	0,39	0,09
O1 (+3.51 eV)	2,3	2,9	1,4	0,5	1,36	4,45	3,27
O2 (+4.50 eV)	32,4	21,2	8,5	8,7	2,66	1,64	0,61
O3 (+4.97 eV)	33,2	21,7	34,9	25,7	2,67	2,27	0,85
O4 (+5.30 eV)	1,1	2,2	20,2	6,6	0,83	5,16	6,19
	**Signal intensity (%)**				**Surface (150 eV) to bulk (300 eV) signal ratio (each peak referred to bulk peak intensity)**		
	**sulfide 150 eV**	**sulfide 300 eV**	**anneal 150 eV**	**anneal 300 eV**	**sulfide**	**anneal**	**(anneal**/**sulfide)**
B (0 eV)	25,9	48,3	35,7	56,8	1,00	1,00	1,00
I1 (−0.18 eV)	1,3	0,6	2,7	2,9	3,74	1,49	0,40
I2 (+0.30 eV)	0,3	0,0	0,0	0,0	-	-	-
O1 (+3.51 eV)	5,1	2,4	1,6	0,2	3,94	12,31	3,12
O2 (+4.50 eV)	39,7	23,5	12,1	18,5	3,15	1,04	0,33
O3 (+4.97 eV)	25,7	21,6	47,3	20,5	2,21	3,67	1,66
O4 (+5.30 eV)	2,1	3,6	0,6	1,0	1,10	0,97	0,88

Next two (“native” or “sulfide” and “anneal”) express the relative average proximity of the state to the surface, higher number indicating closer to the surface, and the last column (“native/anneal” or “sulfide/anneal”) the change in average distribution due to annealing, >1 indicating shift towards the surface and <1 towards the bulk. Upper panel represents the values for the native oxide sample and bottom panel for the sulfide treated sample.

Next we present the effects of annealing observed in P 2*p* on the native oxide sample. From the left panel of Fig. 1 as a function of annealing, one can see an increase in the I1 intensity, suggesting that some of the O-P bonds are reduced into interface related chemical state such as P dimers. This is consistent with the changes in the depth distribution of I1 and O1: the depth distribution of I1 stays relatively constant; *i*.*e*., close to the interface, while the average depth of O1 moves closer to the outer surface because of loss of such P species near the bulk InP (note that when we talk about movement, it could mean either diffusion, or, a reconfiguration of chemical bonds differently on the surface and on the bulk side, resulting in a movement of the average depth of a given state). Another prominent difference is that the intensity of O4 is tremendously increased, and its depth distribution moves toward the surface while O2 has moved deeper and decreased, and O3 remained in its average depth distribution and increased. We note that if the effects observed were due to oxide growth as ‘thickening’ with no chemically induced redistribution, each of the components should be observed moving towards the outermost surface, since they are referred to bulk signal depth. However, we see the effects in both directions without suppression in the bulk peak signal intensity, indicating that the effects are indeed due to local and, eventually, extended conversion of one compound into another at different depths, or possibly also cross-diffusion of different species. To recapitulate, the amount of the highest BE component in the oxide film increases, and its presence as well as increase is more pronounced close to the surface. The O1 component most likely represents an unstable phase at the interface dissociating into P and O. These atoms can further form P-P bonds at the interface as well as more highly oxidized P-O above this region.

The effects of sulfide treatment are observed as increased bulk emission after anneal, less decomposition of O1 into I1 tentatively assigned to P-P bonding at the interface, and distinct conversion of all oxidation states more uniformly into O3 state (+4.97 eV) instead of O4 (+5.30 eV), especially near the surface. A consistent model explaining this effect will be discussed after computational analysis regarding CLSs of interfacial native oxide models.

### In 3*d* measurement

**Figure 2:**
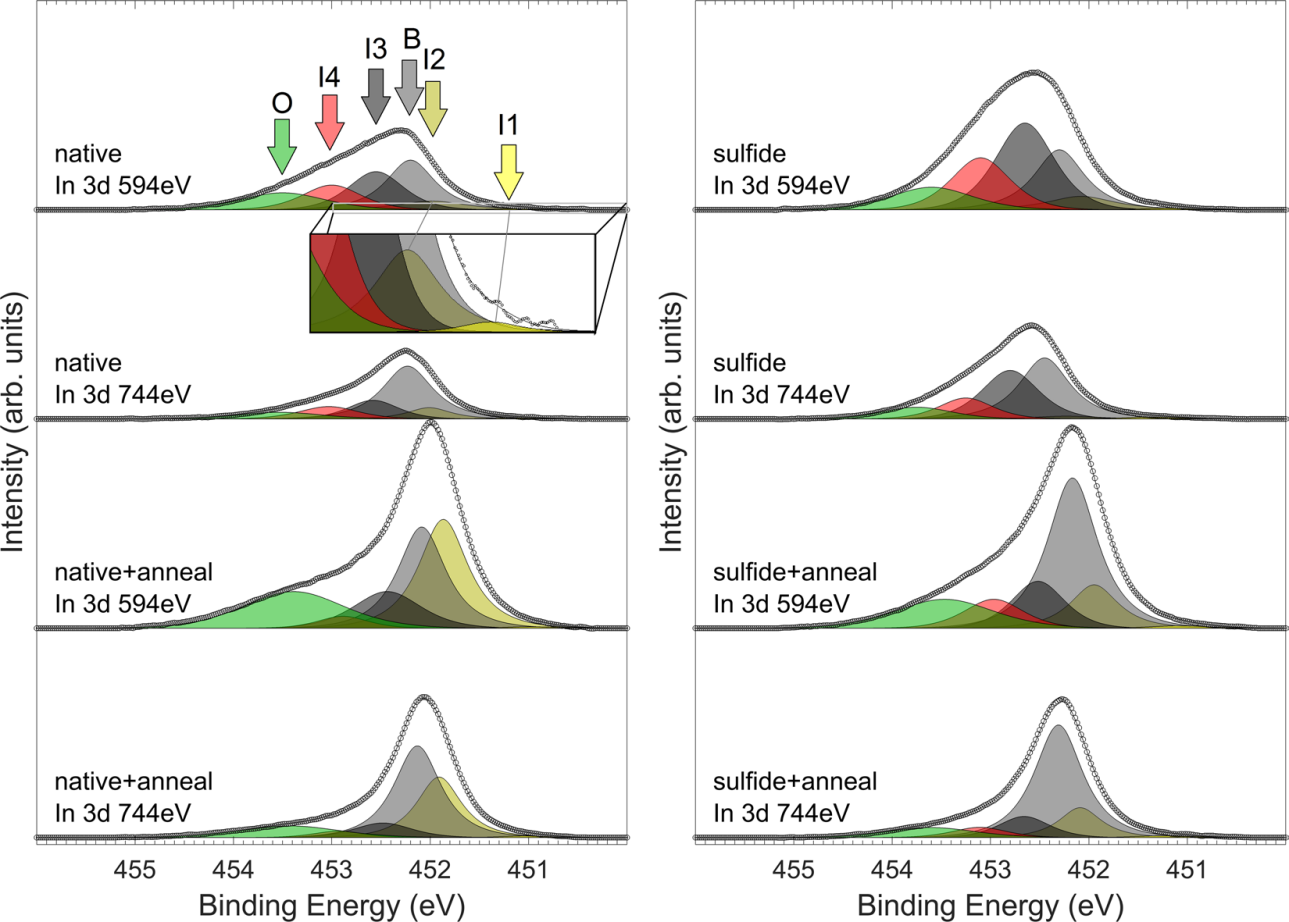
In 3*d* spectra with the fitted peaks. On the left side, sulfide treated sample with corresponding experiments are shown. On the right side, corresponding spectra of sulfide treated sample are shown. The energy label shows the chosen hν of the photons. It is noteworthy that I2 emission is observed for all measurements, yet increased dramatically for native + anneal sample.

In Fig. 2 it can be seen that the intensity of component B in the In 3*d*_3/2_ spectra increases in relation to the other components when the bulk sensitivity of the measurements is increased. Thus, B is straightforwardly interpreted as the component arising from the emission of the bulk crystal chemical state, and its intensity is bound to vary similarly as for P 2*p* B, as described previously. Further justification for the exact position of the bulk component is gained from only slight variation in the BE position (<0.3 eV for a given sample). Table 2 shows the proportional integrated signal intensities of each peak for all of the measurements.

**Table 2:** First columns (under “Signal Intensity”) give the proportional intensities of the fitted peaks for In *3d*_*3/2*_ for each measurement.

	Signal intensity (%)				Surface (150 eV) to bulk (300 eV) signal ratio (each peak referred to bulk peak intensity)		
	native 150 eV	native 300 eV	anneal 150 eV	anneal 300 eV	native	anneal	(anneal/native)
B (0 eV)	31,8	50,7	29,5	46,6	1,00	1,00	1,00
I1 (−1.10 eV)	0,7	0,9	0,5	0,8	1,24	0,90	0,73
I2 (−0.22 eV)	7,7	9,3	32,6	29,4	1,32	1,75	1,33
I3 (+0.35 eV)	25,2	16,4	13,7	8,4	2,45	2,58	1,06
I4 (+0.80 eV)	17,4	12,7	3,4	3,3	2,19	1,63	0,75
O (+1.30 eV)	17,2	9,9	20,3	11,6	2,76	2,76	1,00
	**Signal intensity (%)**				**Surface (150** **eV) to bulk (300** **eV) signal ratio (each peak referred to bulk peak intensity)**		
	**sulfide 150** **eV**	**sulfide 300** **eV**	**anneal 150** **eV**	**anneal 300** **eV**	**sulfide**	**anneal**	**(anneal**/**sulfide)**
B (0 eV)	23,1	40,2	46,9	59,8	1,00	1,00	1,00
I1 (−1.10 eV)	0,6	1,3	0,9	1,1	0,74	0,96	1,30
I2 (−0.22 eV)	6,9	2,5	12,9	13,8	4,81	1,19	0,25
I3 (+0.35 eV)	35,4	32,7	14,0	10,7	1,88	1,67	0,89
I4 (+0.80 eV)	21,3	13,4	9,6	5,6	2,77	2,19	0,79
O (+1.30 eV)	12,7	9,8	15,8	8,9	2,25	2,25	1,00

Next two (“native” or “sulfide” and “anneal”) express the relative average proximity of the state to the surface, higher number indicating closer to the surface, and the last column (“native/anneal” or “sulfide/anneal”) the change in average distribution due to annealing, >1 indicating shift towards the surface and <1 towards the bulk. Upper panel represents the values for the native oxide sample and bottom panel for the sulfide treated sample.

It is clear from Figs 1 and 2 that the In emission changes much more than the P emission due to the annealing. This is consistent with the bond formation energetics^32^: P-O-P and In-O-P bonding configurations are stronger than pure In-O-In. Thus, InP appears to be an exception among various III-V crystals because often the oxidation of group-III elements (*e*.*g*., In) leads to a more stable oxide phase than the group-V (*e*.*g*., As) oxidation^33^.

The other components, I1, I2, I3, I4 and O were introduced to accommodate all the spectral features observed as a function of surface sensitivity and/or different treatments. These same components are fitted to all of the spectra even though we note that some components might be attributed to totally different chemical states or compounds due to different treatments. The variation has not been fitted as separate components, due to a finite resolution, but is rather taken into consideration as inhomogeneous broadening (FWHM) of chemical states (*i*.*e*., there is no separate peak for e.g. In-S as compared to the native oxide sample due to the close proximity of existing peaks). A highly noteworthy observation is, that even though the B peak BE is carefully considered and adjusted, I2 with a negative CLS persists for each of the results.

To study the depth distribution of each emission component, the intensity values of the In 3*d* components are listed in Table 2, similarly to P 2*p* in Table 1.

Before the annealing the origin of I2 is close to the bulk boundary while I4, I3 and O lie increasingly closer to the outer surface. However, during the annealing the depth distribution changes, so that the average depth of the I2 signal moves closer to the surface. All the other interface related components stay fairly still in terms of depth distribution. The proportional bulk intensity remains similar before and after annealing, meaning that there is no significant net segregation towards the surface (that would result in lower proportional B signal intensity), or alternatively that there is significant concomitant evaporation of In species from the surface. The I3 and I4 components quite well retain their depth distribution close to the bulk, meaning that they are likely related to the bulk-native oxide interface. This can also be true for the origins of I2 if there are two overlapping components with a very small difference in BE around the I2 position. I3 and O closely match with the BE shifts reported previously for chemical states of oxide In^+3^ and InPO_4_, respectively [*e*.*g*.^30,31^]. The oxidation state In^+3^ has been commonly fitted with a relatively large peak width to take into account of the commonly observed inhomogeneity in bonding environment of an amorphous native oxide^30,31^. However, in this work we have used quite narrow peak width due to a clear variation in the spectral shape that could not be described using a single peak with the Gaussian emphasis. In contrast, the two separate components, I3 and I4, have been introduced, while the peak O has been fitted with a broad shape. The observation of three separate components reflects the fact (discussed further in the next section) that the same oxidation state, sometimes coarsely attributed as In^3+^ can be contained within several markedly different compounds, and the exact BE shift is dictated by the specific constituents and bonding environment. The large shift of component O from the bulk peak offers an additional support for the bulk peak position by fixing its shift to 1.2 eV so that the shoulder-like feature is well fitted and bulk peak intensity variation is still well explained as a function of surface sensitivity and similar behavior as in P 2*p*.

It is interesting that the emission at the negative BE side (i.e., I2 component) greatly increases during the annealing treatment. Negative CLSs in the group-III spectra of insulator/III-V junctions are typically interpreted as metallic group-III atoms or clusters/droplets and/or filled dangling-bond states. Intuitively, the I2 component’s signal depth and its variation due to annealing (Table 2) suggests that the I2 origin is In atoms detached from the native oxide and diffused into the HfO_2_ film toward the surface. I3 and I4 seem like native oxide components, decomposing somewhat during annealing, and possibly reconfiguring into states corresponding to either I2, or O component that is interpreted to be found at the boundary of native oxide and HfO_2_ according to our straightforward analysis.

Very similar trends are found for the sulfide treated sample. However, increase in the proportion of B emission is significantly higher (as dictated by the similar trend in P 2*p*), and there is a more significant decrease in the initially higher I3 and I4 components; these components are likely related to In-S bonding sites at the interface area, and overlapping with oxide peaks; both In-S and native oxide peaks are tentatively assigned to I3 and I4. Moreover, as they are significantly reduced due to annealing while bulk-emission increases, it is suggested that S-containing interface transforms into a more abrupt barrier between InP and HfO_2_, leaving less dangling bonds as described below, and seen also here as more intense B signal due to more ideal reconfiguration beneath the oxide. Lower increase in I2 and O suggests also less detachment of In to diffuse and/or reconfigure in the HfO_2_ film, consistent with earlier literature^19^. Some In could still diffuse to the surface from the interface, but a more limited supply will result in much less observed In on the surface, especially after prolonged annealing as In will most likely also evaporate when reaching the surface. This effect is also consistent with a significantly higher proportional increase of B signal after annealing.

### Calculated bulk oxide and interface core-level shifts

The In 3*d* and P 2*p* relative core-level binding energies of several common bulk oxides of In and P were calculated, and the results are presented in Table 3. The calculational results reveal interesting trends and set important reference values for oxidized semiconductor systems. The relative binding energies are represented with respect to the Fermi level (not the vacuum level), which is the common practice in experiments. The In 3*d* and P 2*p* core-level binding energies in InP are set to zero. Therefore, positive (negative) CLS means increased (decreased) BE.

**Table 3:** The experimental and calculated In 3*d* and P 2*p* relative binding energies in In_2_O_3_, InPO_4_, In(PO_3_)_3_ and P_2_O_5_.

	In 3*d*	P 2*p*
**In**_**2**_ **O**_**3**_		
Exp.	0.1–0.3	
CS (IS)	−1.23 (−1.35)	
CS (IS) 4*d*	−1.08 (−1.71)	
**InPO**_**4**_		
Exp.	1.0–1.3	5.2–5.3
CS (IS)	0.18 (0.24)	5.21 (2.13)
CS (IS) 4*d*	0.17 (−0.27)	4.78 (2.13)
**In(PO**_**3**_ **)**_**3**_		
Exp.	1.8	6.2
CS (IS)	0.57 (0.48)	6.91 (3.19)
CS (IS) 4*d*	0.58 (−0.12)	6.46 (3.20)
**P**_**2**_ **O**_**5**_		
Exp.		6.8–7.5
CS (IS)		7.53 (4.23)

The calculations were done within the complete screening (CS) and initial state (IS) models. The In 4d states were core electrons or valence electrons (4d). The experimental values are from refs^58–60^. The experimental In and P binding energies in InP are equal to 444.4 eV and 128.8 eV^58,60^.

The P 2*p* relative BEs or CLSs within the complete screening (CS) model are in a relatively good agreement with the experimental ones. However, the disagreement is larger for the In 3*d* CLSs, which are underestimated by about 1.0–1.2 eV, if the In 4*d* electrons are treated as valence electrons. Still, the experimental trend in the In 3*d* CLS shown in the Table 3 is reproduced by the calculations. Obviously, there are many potential sources of errors both in the calculations and experiments. Concerning calculations, in particular, the hybrid Heyd-Scuseria-Ernzerhof (HSE) density functional^34^ and homogeneous background charge as a replacement for the additional neutralizing electron in the complete screening calculation^35^ were tested. These methods did not change the In 3*d* or P 2*p* CLS significantly. It should be noted that the CLS are usually calculated for systems, like surfaces or impurities, which can be modeled by a single geometrical construction. This increases accuracy significantly.

Several remarks can be made. The P CLSs are much larger than the In CLSs. The P valence charge is strongly bound due to the increased nuclear charge (which is not compensated by the increased electronic repulsion), and therefore, the electronic charge transfer in the ionic bond leads to larger CLS. Experimental core-level shifts are indeed often interpreted intuitively in terms of the transferred valence charge, see *e*.*g*. ref.^36^. It is commonly assumed that charge transfer increases with the ionicity of the bond. Therefore, ionic bonds should induce larger charge transfer than covalent bonds do. The In and P atoms lose electronic charge in oxides which increases the binding energies of the In and P core states. However, this is obviously not the whole story, because the In 3*d* initial state model CLSs (calculated with In 4*d* states in valence) are negative especially in the In_2_O_3_ ionic oxide. The CLSs are often interpreted more quantitatively in terms of the oxidation state which is occasionally even identified with the number of nearest neighbor O atoms, because this interpretation is valid for the SiO_2_/Si interfaces^6^. However, In and P have oxidation states of +3 and +5 in all compounds considered in the Table 3. Furthermore, the In and P atoms occupy octahedral and tetrahedral positions, respectively, in all considered oxides. Still, the In or P CLSs are significantly different in various compounds. It can be noted that the CLSs become larger as the oxygen concentration increases. The second nearest neighbor configuration is also changed with the composition. Ionization generally increases the CLS in relative to the initial state model CLS, and this effect is much stronger for the P CLSs than the In CLSs. The discrepancies between the experimental and calculated complete screening CLSs might be tentatively contributed to the non-complete screening in the experiments.

The CLSs of the In and P impurities in the HfO_2_ are −0.18 eV and 6.17 eV, respectively. The corresponding initial state CLSs are 0.17 eV and 4.19 eV. An interfacial atom can have a different (nearest) neighbor atomic configuration than the bulk atoms have. Furthermore, the interface dipole may affect the CLS. Different HfO_2_/InP interface models were constructed to investigate, how the CLSs depend on the atomic environment. A semi-coherent model (O10), which has a relatively small lattice mismatch and which does not show interface states in the band gap, was introduced for the HfO_2_/GaP and HfO_2_/GaAs interfaces^37,38^. The lattice mismatch of this model can be kept relatively small for the HfO_2_/InP interface by replacing the simple tetragonal HfO_2_ (space group 137) used in the Ref.^37^ in the O10 model with the anatase HfO_2_ (body-centered tetragonal; space group 141)^37,39^. It should be noted that the CLSs of the In and P impurity atoms do not depend on the chosen HfO_2_ phase. The impurity CLSs calculated for the bulk ground state monoclinic structure are practically identical to those calculated for the anatase structure. Nine different HfO_2_/InP interface models based on the O10 model were constructed to investigate the CLSs of the impurity and interfacial atoms. The InP part can be either In or P terminated whereas the In and P concentrations vary at the first layer of the HfO_2_ part. There are many different kinds of In and P atom environments at the interface due to these variations. The first layer of the InP part is dimerized and oxygen atoms can be inserted into the dimers. All considered interfaces have an energy gap which points out that semi-coherent interfaces are electronically flexible. The electron counting rule (ECR) can be satisfied by In unoccupied and P occupied dangling bonds. The complete screening CLSs of the P atoms can be classified in terms of the close O neighbors (*i*.*e*., O bonds) which is shown in Table 4 (only complete screening CLSs are considered below). This is very simple and interesting taking into account that some of the interface atoms have an unusual atomic neighbor configuration. Furthermore, the relative valence band offsets vary within an interval of 1.5 eV. The robustness of the classification suggests that the same principle might be applied also to different kind of interfaces (*e*.*g*., more diffuse interfaces). It should be noted that the concept of “close O neighbor” is convenient in practice. Taking into account the calculated As 2*p* CLSs at the Al_2_O_3_/GaAs interfaces^40^ (the only previous first-principles study of oxide/III-V interface CLSs found) it is possible that similar interpretation might be valid for III-V semiconductors more generally. It should also be noted that the P CLSs are not increased significantly by increasing the number of close O neighbors from four.

**Table 4:** The P 2*p* and In 3*d* complete screening (CS) and initial state (IS) model CLSs of several compositionally different semi-coherent HfO_2_/InP interfaces grouped in terms of the close O neighbors.

N_O_	CS P	IS P	CS In	IS In
0	0.17–0.49	0.28–0.42	−0.41–0.20	−0.16–0.33
1	0.48–2.03 (1.10–2.03)	0.18–1.05 (0.71–1.05)	−0.58–0.52	−0.39–0.95
2	2.10–3.06	1.50–2.87	−0.39–0.47	−0.31–0.67
3	3.05–4.48	1.00–3.15	−0.38–0.46	−0.19–0.24
4	5.56	3.00	−0.63–0.66	−0.38–0.39
5	5.77	3.64	−0.84––0.33	−0.16–0.25
6			−0.10	0.12

A P (In) atom has a close O neighbor, if the interatomic distance is smaller than 2.0 Å (2.7 Å). The chosen cutoffs are somewhat arbitrary (as the concept of bond), but the found trends are not affected by this slight arbitrariness. One P + 1 configuration is considered unlikely (having two relatively distant Hf neighbors in addition to one O neighbor). The parenthesis show values without this configuration. There is only one value for the P N_O_ (number of close O neighbors) equal to four and five. These P atoms are above the interface layer and substitute Hf atoms. Similarly there is only one value for the In N_O_ equal to six.

However, it is shown in the Table 4 that there is no correlation between the CLSs and the number of close neighbors of the In atoms. This shows that the charge transfer model expressed in terms of the number of oxygen neighbors is not generally valid assuming that the calculated results reproduce the experimental trends. It has been shown that the local charge or *d* charge of transition metal atoms is approximately constant in different “charge states” in several oxides^41,42^. However, the phenomena expressed in terms of the “oxidation state” or “charge state” are real in these cases^41,42^.

An interesting correlation was found between the composition of the interface layer at the boundary of the HfO_2_ oxide part and the band offset. The valence band maximum of the oxide part seems to decrease with respect to the InP part with the concentration of the P atoms within the oxide interface layer. This might be attributed to the occupied P dangling bonds which cause gap states. The band offsets reported by Santosh *et al*. for a different O10 model (In terminated interfaces were not considered) seem to follow roughly this trend^22^. The band offsets are reflected also in the CLSs of the substitutional/impurity In and P atoms within deeper layers of the HfO_2_. The CLSs for the substitutional In and P in the centre of the HfO_2_ part within the O10 model are −0.89 eV and 5.49 eV. The magnitudes of these CLSs are smaller than those calculated for pure separate HfO_2_. The decreased CLSs imply that due to the interface there is a band offset which decreases the CLS within the HfO_2_/InP interface system relative to the separate bulk HfO_2_ and InP phases. The band offset is larger for other interface models within an interval of 1.5 eV increasing the CLS.

It is possible that there is some native oxide between the HfO_2_ and InP parts, and this is the most realistic scenario for the investigated structures especially since HfO_2_ was specifically grown on a native oxide on one of the samples. Two InPO_4_ model oxides, constrained to the InP interface area, are introduced to calculate CLSs for two coherent HfO_2_/InPO_4_/InP double interface systems. The first model oxide (InPO_4_-a) is based on the HfO_2_ anatase structure in which every second atomic layer in direction of the longest lattice parameter is substituted with either In or P atoms. However, the doubling of the length of the *c* lattice parameter decreases total energy as the PO_4_ tetrahedra can be oriented in different ways. The second oxide model structure (InPO_4_-b) can be relaxed from an orthorhombic initial structure (by non-symmetric atomic displacements). The space group number of this initial structure is 80. There are two O atom Wyckoff positions (8a) (*x* = 0.232; *y* = 0.301; *z* = 0.297; *x* = 0.778; *y* = 0.160; *z* = 0.160). The Wyckoff *z* parameters for the In and P (4*a*) positions are 0.697 and 0.098, respectively. The resulting InPO_4_-a and InPO_4_-b oxide structures may depend on the size of the chosen cell due to disorder. The structures used are calculated for an (2 × 2) interface area. The unrelaxed model oxides are composed of similar structural motifs with different stackings. Total energies of these oxides are larger than the total energy of the ground state InPO_4_ structure. The total energies of the InPO_4_-a and InPO_4_-b are 0.17 eV/atom and 0.09 eV/atom with respect to the total energy of the ground state InPO_4_ (orthorhombic lattice, ref.^43^). For comparison, the only bulk InPO_4_ phase with a square face, scheelite^43^, has a relative total energy of 0.10 eV/atom. Therefore, the total energies of the considered model oxides are not unrealistically high. Furthermore, the decreasing of the interface area increases the total energy of these model InPO_4_ oxides in opposition to the HfO_2_. Therefore, it is not relevant to consider semi-coherent InPO_4_/InP interfaces based on the O10 model. The bulk complete screening P 2*p* CLSs (In 4*d* states in core) for the InPO_4_-a and InPO_4_-b are 5.02 eV and 4.55 eV, respectively. The corresponding values for the ground state and scheelite InPO_4_ are 5.21 eV and 5.27 eV.

The InPO_4_-a and InPO_4_-b oxides may grow in thin films, if the interface energy for the ground state InPO_4_ structure is relatively high. The total energy of the double interface system with an InPO_4_-a thin film (two In-P oxide double layers) is slightly lower than that based on the InPO_4_-b [0.26 eV per (1 × 1) interface area], which points out that the interface energy is relatively low for the InPO_4_-a structure. The result also shows that a relatively significant bulk InPO_4_ total energy difference (~0.1 eV/atom) may be compensated by the interface energy difference in thin films. It is supposed that the chosen terminations of the InPO_4_ models are energetically favorable due to the characteristic form of the PO_4_ tetrahedron found in all InPO_4_ phases (no broken PO_4_ tetrahedra).

**Figure 3:**
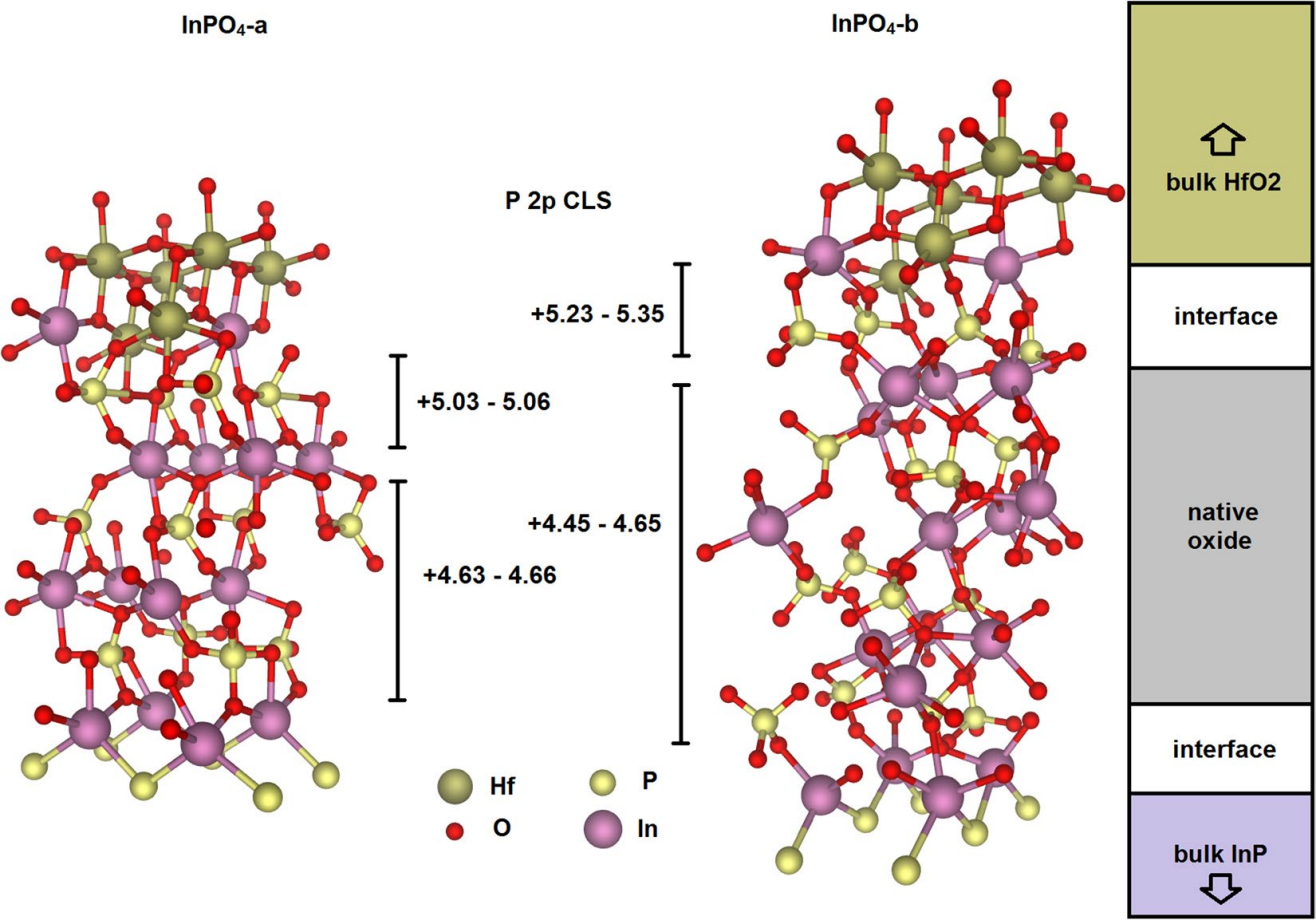
InPO_4_-a and InPO_4_-b structures between bulk InP and HfO_2_ shown with the calculated native oxide CLSs of P 2p of the corresponding structures (eV).

The CLSs for the double interface systems were calculated using from two to four In-P oxide double layers. The highest P CLSs originate just below the HfO_2_, while the other P layers in the InPO_4_ show quite similar CLSs among each other. The P CLSs for the interface layer just below the HfO_2_ and other InPO_4_ layers are 5.03–5.06 eV and 4.63–4.66 eV, and 5.23–5.35 eV and 4.45–4.65 eV for the InPO_4_-a and InPO_4_-b, respectively. The native oxide and interface regions of this unit cell structure are shown in the Fig. 3. The CLSs are quite similar, although the difference between the bulk InPO_4_-a and InPO_4_-b CLSs is slightly larger. The valence band offset is smaller for the InPO_4_-a, which decreases the CLSs for the InPO_4_-a. The orientation of the PO_4_ tetrahedra are different in the InPO_4_-a and InPO_4_-b, which may contribute to the relative band offset. Thus, band bending is possible also without composition changes within the interfacial layers at the oxide and semiconductor parts. The results point out that different In and P CLSs may be originated from a chemically uniform interface system. Thus, the different experimental CLSs do not originate necessarily from different oxide films having, *e*.*g*., In_2_O_3_ and InPO_4_ compositions.

## Discussion

Finally, the experimental CLSs are analyzed using the calculated CLSs. The experimental P CLSs (I1, I2, O1, O2, O3, O4) are −0.18, 0.30, 3.51, 4.50, 4.97, 5.30 eV. It is noted first that the experimental P CLSs are in good agreement with the calculated ones for the model InPO_4_/InP interfaces. The results show that P oxidation states +1 and +2 are missing. This suggests that the first interface layer in the InP part is composed of In atoms, because the P dimers probably tend to be oxidized. However, if the interface includes P-P dimers, they cause small positive shift 0.2–0.5 eV for P 2*p* according to the calculations. The O1 peak (3.51 eV) vanishes with annealing which means that the broken PO_4_ tetrahedra disappear (Table 4). The relative intensity of the O2 (4.50 eV) is decreased whereas the relative intensities of the O3 (4.97 eV) and O4 (5.30 eV) are increased by annealing which could reflect thinning of the InPO_4_ part (because then the relative weight of the layer just below the HfO_2_ increases), but the depth analysis gives reason to suspect other effects than just thinning. On the other hand, the relative amount of different InPO_4_ phases could be changed. Alternatively, when considering the effect of previously observed indium out-diffusion^18^, it is likely that composition also changes. A noteworthy observation about InPO_4_ CLSs is that all of the native oxide stacks considered produce smaller shifts in the mid-layer of the native oxide than the corresponding bulk oxide (about 4.6 eV for InPO_4_-a and InPO_4_-b vs. 5.2 eV for InPO_4_ bulk). Thus, it is possible that out-diffusion could cause In-deficient phases in mid-layers of the native oxide (originally mainly composed of InPO_4_) similar to In(PO_3_)_3_ (6.2–6.9 eV in bulk), that would match the O4 BE (5.3 eV). This is consistent with the higher stability of P-O bonding as compared to In-O^18^. Since there is out-diffusion of In in the HfO_2_, the depth analysis is well reasoned: O4 is observed an increase especially further away from bulk than other components, probably because the rate of out-diffusion is likely higher closer to the native oxide/HfO_2_ interface.

Furthermore, the sulfide treatment has been observed to suppress the indium out-diffusion^18^. In our experiments and based on the above analysis, this is observed in P 2*p* as the lack of O4 signal, or In-deficient bonding, consistently with the amount of In staying relatively constant in the sulfide/native oxide film. The O3 component intensity increases, which is likely related to the increased relative weight of the layer just below the HfO_2_ as described previously. Here, also thinning of the sulfide/native oxide film is plausible, since the proportional emission of B signal increases after annealing.

In order to justify the analysis above, similar effects need to be observed also for In. However, it is to be noted that our computational results underscore the difficulty in making well justified interpretations about the In 3*d* XPS results for our samples, as the CLSs are found with only slight offset from the bulk core-level. Furthermore, the shifts are not consistent with the amount of nearest-neighbor O, or straightforwardly with valence charge, as opposed to P 2*p*. However, the relative differences between BEs of different In-P oxide bulk phases are close to the ones reported in literature. In_2_O_3_ is however typically associated with positive shifts, contrary to the computational results. On the other hand, reference data tables suggest very similar BEs for bulk In_2_O_3_ and InP^44^, which is why a small negative shift for In_2_O_3_ in the structure for any particular oxide/InP systems is not beyond reasoning, but on the contrary, suggested also by the calculations. Without taking this into consideration, there is a considerable chance of misinterpretation since, as mentioned, elemental/metallic In can cause very similar shifts.

The out-diffusion of indium being the established culprit of device performance degradation on HfO_2_/InP interfaces, it is of necessity to consider this effect as has been done above. The defective sites accountable for the diffusion (interstitial defects containing In)^45^ are not, however taken into account in the spectral analysis. A concentration of these defects that would be detectable in XPS (0.1–1%) would also significantly alter the oxide characteristics and cause much higher amount of trap states that has been observed^3^. However, despite a small concentration, a significant amount of In can diffuse to the surface if the flow is continuous as can be assumed during the annealing. This has been observed previously with LEIS^4^, and thus, surface segregated In needs to be considered as a chemical state observable especially in the surface sensitive XPS setup such as has been utilized here.

Based on the analysis for P 2*p* above, and the fact that stoichiometrically identical compound can cause markedly different CLSs, we assign the In 3*d* peaks I3, I4 and O to InPO_4_ and In(PO_3_)_3_ contained within the native oxide or at the interface of HfO_2_. The concentration of InPO_4_ [I3 and I4] decreases as In(PO_3_)_3_ [O] bonding increases near the interface boundary after annealing, consistent with the analysis above. The depth distributions stay fairly constant, which is consistent apart from In(PO_3_)_3_ which changes much more towards the surface for P 2*p*. This could be because, the stoichiometric factor of In in In(PO_3_)_3_ is smaller than that of P, so that similar change in concentration has only a third of an effect on the intensity of the corresponding component, and because there is likely overlapping of states related to either interface bonding or other oxide phases, that do not change their depth distribution, and thus diminish the effect of changes in In(PO_3_)_3_ seen in depth distribution. The I2 is increased dramatically and brought closer to the surface after the annealing. However, the depth distribution is not changed as dramatically, indicating that there is increase in the concentration of this component both near the bulk and the surface. Here the previous reports and our complementary computational results bring insight onto identification of the state; I2 actually likely consists of two overlapping peaks: In_2_O_3_ type emission that is observed near the bulk, and elemental/metallic In at the surface due to out-diffusion. Some of the segregated In is evaporated, which is observed as an increase in the B signal relative to the others.

Noting the formation of In_2_O_3_, we suggest a following model which accounts for all of the effects discussed above. Initially the native oxide film or the thin oxide present also in sulfide-treated sample consists mainly of InPO_4_. During annealing, the P atoms tend to bond with O, producing In(PO_3_)_3_, or other In-deficient phases. For P, the most prominent differences are at the topmost layers of the native oxide, indicating either relatively more significant proportion of P atoms having multiple bonds to the O in HfO_2_ layer, or more prominent formation of In(PO_3_)_3_. This relieves In, that can form In_2_O_3_ or other In-rich phases locally, causing some phase separation. Some of the O and/or In atoms could be provided by phase separation at the oxide/InP interface into P dimers. On the other hand, atomic In in the upper layers of native oxide is able to diffuse to the surface through vacancy sites in HfO_2_. This makes sense, since as noted above, the most marked differences are at the topmost layers of the native oxide, and the differences are due to more P-O bonds. It is possible that during annealing in the HfO_2_ film, extra O vacancies are formed, through which In has been reported to diffuse. Thus, the degradation occurs due to the inherent chemistry of the native oxide film as a result of these synergistic effects, and thus, it is readily prevented by saturation of InP surface dangling bonds with e.g. S after the sulfide treatment. However, being an *ex-situ* method, the sulfide passivation is not able to prevent the formation of bonding observed in the native oxide altogether, which is why similar effects are seen on the sulfide-treated sample, but to a lesser extent.

## Conclusions

The presented results for HfO_2_/InP junctions demonstrate that the semiconductor oxidation can cause negative CLSs (*i*.*e*., a decrease in core-level BE as compared to the clean semiconductor), in contrast to the common hypothesis that the material oxidation causes positive CLSs, which is based on the charge-transfer model and the well-understood SiO_2_/Si system. The P CLSs can be estimated robustly at the abrupt HfO_2_/InP interfaces considering the number of close O neighbors irrespective of the other atomic neighbors, resembling the SiO_2_/Si system, but no similar correlation was found for the In CLSs. The In CLSs cannot be explained by the number of close O neighbors. The results emphasize that the special care needs to put on determining the reference BE (e.g., bulk emission peak position) by changing the surface-sensitivity of the measurements.

To strengthen the XPS analysis and to utilize full potential of the method, we have combined *ab initio* calculations and synchrotron XPS in the study of the example case of HfO_2_/InP. Two model structures for the InPO_4_/InP were introduced. These are the first model interfaces structures for native oxides of III-V semiconductors which can be used, *e*.*g*., to estimate, whether coherent or semi-coherent interface growth is preferred. A correlation was found between the number of P atoms in the interface oxide layer and the band offset at the semi-coherent HfO_2_/InP interfaces. A model consistent with our experiments and calculations as well as previous reports concerning annealing effects on HfO_2_/InP system has been presented. We suggest that annealing can induce effects at the oxide/semiconductor interface that result in CLSs without necessarily changing the chemical stoichiometry, but rather the bonding configuration. Furthermore, markedly different chemical states can be observed at the same BE. These effects complicate XPS analyses, and the results underline the importance of complementary studies and high resolution XPS data. Here, we have been able to identify the atomic origins of CLSs that can remain totally hidden in the traditional laboratory XPS spectra. Our findings may pave the way for systematic improvement of the interpretation of CLS in relation to characterization of materials at the atomic scale both in academic and industrial investigations where CLS are at the heart of advancing knowledge.

## Methods

### Sample and measurement setup

Our XPS experiments were carried out in the synchrotron radiation centre MAX-lab, Lund, Sweden, at beamline I311. The base pressure of the experimental station was in 10^−10^ mbar range. The photon energy, *hν*, was varied to measure the P 2*p* and In 3*d* peaks with two different kinetic energies (KE, i.e., surface sensitivities): 150 eV and 300 eV (*hν* of 279 eV and 429 eV for P 2*p*, and 594 eV and 744 eV for In 3*d*). Gaussian broadening of the signal arising from the instrumentation is estimated to be less than 0.15 eV. Two samples were investigated. An InP(100) crystal with a native oxide film on top of which a HfO_2_ film was grown by ALD. Another InP(100) sample was treated by 10% (NH_4_)_2_S aqueous solution diluted from 20% aqueous solution. TDMA-Hf was used as the metal precursor, and H_2_O vapor as the oxidant precursor with ultrahigh purity N_2_ gas as the carrier gas. The temperature of ALD was at 250 °C, and 20 cycles of ALD of HfO_2_ were grown on the InP wafer by a pulse sequence of Hf/purge/H_2_O/purge for 0.1 s/10 s/0.1 s/10 s, respectively. The growth corresponds to a uniform film thickness of approximately 1.6 nm with an established growth rate of 0.08 nm per cycle^18^. After the ALD growth the samples were transferred to the *ex-situ* synchrotron radiation centre. The samples were measured before and after post-growth annealing at 400–450 °C in the UHV system, to investigate temperature dependent compositional changes in the oxide film and at the oxide-semiconductor interface.

### Spectral analysis

A fitting procedure similar to one used for describing surface CLSs for In containing semiconductors was applied with CasaXPS software version 2.3.16^46^. However, due to overlapping of In 3*d*_5/2_ with Hf 4*p*_1/2_, In 3*d*_3/2_ was used in the fitting because this peak still gives a high intensity. This approach is convenient due to the relatively high spin-orbit splitting of In 3*d* (∼7.5 eV) so that overlapping of the spin-orbit peaks can be avoided. Fitting was carried out to deconstruct XPS spectra into a minimum number of individual components that were required to reproduce the spectral features observed. An essential requirement in the fitting was that higher *hν*, which provides a more bulk sensitive measurement, caused higher relative intensity for the bulk peaks. Even though bulk peak intensity ratio of P 2*p*_3/2_ to In 3*d*_3/2_ is not 1:1 (due to different photoionization cross-section and photon flux), the relative difference in absolute bulk signal intensity needs to vary identically between treatments for these peaks with a given KE, since bulk bonding consists of In-P bonds only and this bonding environment starts from beneath the exact same depth. Thus, the bulk peak intensity ratio of In 3*d*_3/2_ at a given KE before and after annealing treatment was bound to vary in similar ratio as the bulk P 2*p*_3/2_ at the same KE, by adjusting the bulk peak BE position. 1:1 stoichiometry condition in InP bonding environment was more reliably satisfied this way, since P 2*p*_3/2_ bulk signal was observed without significant overlappings. Variation in the photon flux from the synchrotron ring was taken into account by scaling the intensity of each measurement with the ring current during the corresponding measurement, so that absolute intensity values of different measurements could be reliably compared this way. The actual photon flux from the beamline optics to the sample could not be taken into consideration, but should be similar for two measurements with the same *hν* after scaling with the ring current.

**Figure 4:**
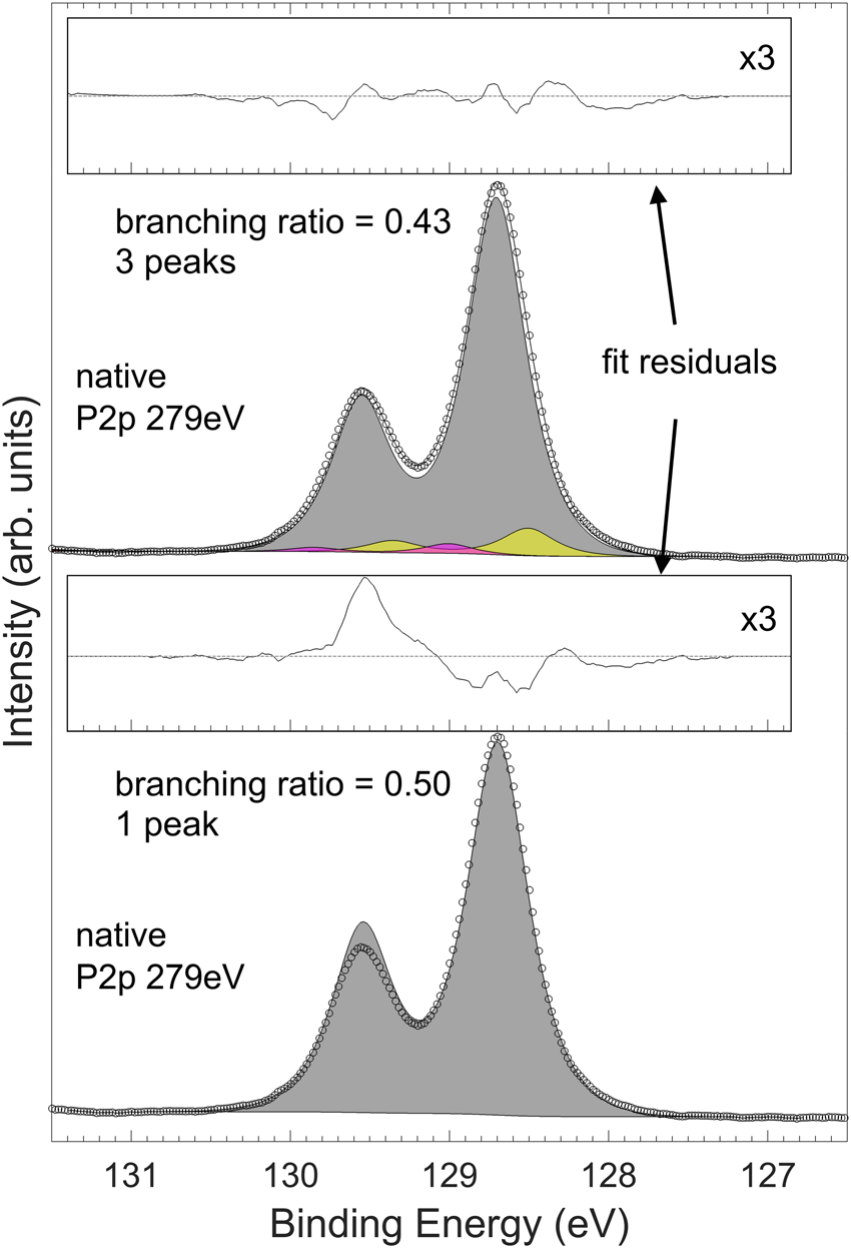
An example P 2*p* spectrum with separate fittings showing the effect of branching ratio parameter and additional components to the fit residual. The applied parameters (top spectrum) result in a low residual especially at branching point and systematically reduced around both P 2*p*_*3/2*_ and 2*p*_*1/2*_ without overemphasizing any features.

The fitting parameters which reproduced the spectral envelopes are shown in Tables 5 and 6. The spin-orbit splitting was 0.85 eV and branching ratio 0.40–0.43 between the P 2*p*_3/2_ and 2*p*_1/2_ peaks. A P 2*p* peak sum with 0.5 branching ratio, that would account for the envelope spectrum could not be introduced, which is obvious from the absence of any significant tail features at higher or lower BE. No other photoelectron peak should be observed at this BE for Hf, O, P, In or C either. Thus, we expect this discrepancy to be caused by some other external effect, such as diffraction or multiplet splitting. Figure 4 illustrates an example of the significant reduction in asymmetric fit residual for P 2*p* bulk peak area when changing the branching ratio and adding two adjacent components. The justifications for the introduced components are further discussed in the Results section.

**Table 5:** Peak fitting parameters for P 2*p*_*3/2*_ components before and after annealing as well as for as-grown and S-treated samples.

	Shape	FWHM (eV)	BE position (eV)
B	GL(80)	0.39–0.45	128.55–128.85
I1	GL(80)	0.35–0.5	B-0.18
I2	GL(80)	0.35–0.5	B + 0.3
O1	GL(65)	0.65–0.85	B + 3.51
O2	GL(65)	0.65–0.85	B + 4.5
O3	GL(65)	0.65–0.9	B + 4.97
O4	GL(65)	0.65–1.0	B + 5.3

BE position of components O1–O4 was allowed to vary 0.1 eV from their fixed position.

**Table 6:** Peak fitting parameters for the In 3*d*_*3/2*_ components.

	Shape	FWHM (eV)	BE position (eV)
B	GL(87)	0.5–0.6	452.05–452.45
I1	GL(60)	0.5–0.7	B-1.1
I2	GL(87)	0.5–0.8	B-0.22
I3	GL(60)	0.6–0.8	B + 0.35
I4	GL(60)	0.6–0.7	B + 0.8
O	GL(50)	0.8–1.2	B + 1.3

A systematic BE increase of 0.1–0.2 eV was observed in all of the S-treated sample’s components. We note that this could be related to charge redistribution near the interface, but such analysis is omitted since the Fermi-energy was not calibrated separately for the measurements of the two different samples.

## Calculations

Calculations were performed using an *ab initio* density functional theory (DFT) total energy method within the Perdew-Burke-Ernzerhof (PBE) generalized gradient approximation (GGA)^47^. The approach is based on the plane wave basis and projector augmented wave method^48,49^ (Vienna *ab initio* simulation package, VASP)^50–53^. The interfaces were modeled using unit cells with two equal (single or double) interfaces without vacuum. The optimization of the atomic structure was performed using the conjugate gradient minimization of the total energy with respect to the atomic coordinates. Atoms were relaxed until the remaining forces were less than 20 meV/Å. The plane wave cutoff energies of 350 eV and 500 eV were used for interface and bulk calculations, respectively. All test calculations with the cutoff energy of 500 eV showed only marginal differences for the interface CLSs and total energies. The In 4*d* and P 3*d* as well as Hf 5*p* electrons were treated as core electrons within the interface systems. The In 4*d* electrons were treated both as valence electrons and core electrons in the bulk calculations. The interface *k* point sampling was carried out by the Monkhorst-Pack scheme^54^ using a 4 × 4 × 1 mesh for (2 × 2) interface area. The origin was shifted to the Γ point.

The HfO_2_/InP interface unit cells consist of 8–9 layers of group III atoms, 8–9 layers of group V atoms, 5 layers of Hf atoms, and 6 layers of O atoms. The HfO_2_/InPO_4_/InP double interface unit cells consist of 10–14 layers of group III atoms, 10–14 layers of group V atoms, 4–6 layers of Hf atoms, and 11–19 layers of O atoms. The In_2_O_3_, InPO_4,_ In(PO_3_)_3_ and P_2_O_5_ initial structures are obtained from the refs^43,55–57^. The initial state CLSs were determined by calculating the electrostatic potential at each ion core. The Fermi level is set to the middle of the band gap. The complete screening calculations (core hole and an extra screening valence electron) were calculated using large supercells (about 100 atoms for bulk calculations) to minimize artificial interaction of the core-ionized atoms. Some test calculations were performed using even larger cells (*e*.*g*., 640 atoms for In_2_O_3_). The atoms in the central layers of the InP part represent bulk atoms in the interface calculations. The inaccuracy of the CLSs with respect to the length of the InP part is assessed to be smaller than 0.1 eV. The interface area is (2 × 2) except for the In and P impurity calculations in which an (4 × 4) interface area was used.

## Supplementary information

Supporting Information

## Data Availability

The photoelectron spectra and data used for the computational studies are available from the corresponding authors on reasonable request.
