# Supplementary material for: Unusual oxidation-induced core-level shifts at the HfO2/InP interface
Source: Sci Rep. 2019 Feb 6;9:1462. doi: 10.1038/s41598-018-37518-2 (PMC6365577; doi:10.1038/s41598-018-37518-2)
Supplement: Supplementary file 1 — Supporting Information [file 41598_2018_37518_MOESM1_ESM.pdf]

Supporting Information

Unusual oxidation-induced core-level shifts at the HfO_2/InP interface

Jaakko Mäkelä[1*], Antti Lahti[1], Marjukka Tuominen[1], Muhammad Yasir[1], Mikhail Kuzmin[1,2], Pekka Laukkanen[1], Kalevi Kokko[1], Marko P.J. Punkkinen[1*], Hong Dong,[3†] Barry Brennan[3‡], Robert M. Wallace[3]

[1] *Department of Physics and Astronomy, University of Turku, FI-20014 Turku, Finland*

[2] *Ioffe Physical-Technical Institute, Russian Academy of Sciences, St. Petersburg 194021, Russian Federation*

[3] *Department of Materials Science and Engineering, The University of Texas at Dallas, Richardson, Texas 75080, USA*

---

[*] *Corresponding authors, email: jaakko.m.makela@utu.fi; marpunk@utu.fi*
[†] *Current address: Department of Electronics and Tianjin Key Laboratory of Photo-Electronic Thin Film Device and Technology, Nankai University, Tianjin, 300071, China*
[‡] *Current address: National Physical Laboratory, Hampton Road, Teddington, TW11 0LW, United Kingdom*

Fig. S1 illustrates effects of annealing on Hf 4*f* emission for native oxide sample (left panel) and S 2*p* emission for sulfidized sample (right panel). The detailed effects in the HfO_2 film are not discussed in the present study as the fitting becomes problematic due to the remarkable overlapping of In 4*d*, making the Hf 4*f* envelope consist of more than 14 individual peaks. The significant increase in emission from In observed in the main article Fig. 2 after annealing is observed also here in In 4*d*. Simple interpretations can be concluded as follows: no shifting of Hf 4*f* towards the Fermi-level is observed, i.e., band bending remains fairly unaffected in the oxide film. Instead, small shift towards higher BE could be present. This could be due to more stoichiometric HfO_2, owing to experimentally observed much higher BE of Hf oxide phases than elemental Hf [44]. Interactions with native oxide film are not excluded, but it should be noted that reduction of HfO_2 into substoichiometric species should be observed as lower BE emission, which is not seen here. Thus, we expect the HfO_2 to be relatively stable in these conditions.

Conclusions drawn in the case of sulfidized sample are discussed here also based on S 2*p* emission. The spectrum envelope is effectively narrowed and pushed towards lower BE, but peak shape is also remarkably changed which is readily observed in the fitting as suppression of highest BE component and increase in the lower BE components. Annealing does not induce change in total intensity, so that S depth distribution is not changed but remains at the interface. Before HfO_2 deposition the S-terminated surface is exposed to atmospheric oxygen, possibly inducing excess O-bonding (S-O-Hf). Based on experimental data [44], we assign S 2*p* highest BE component to S with O and Hf/In/P bonds. Annealing tends to reconfigure the S-bonds to be more highly coordinated towards Hf/In/P (sulfide compounds), seen as lower BE components. Thus, sulfide treated sample contains less native oxide, and also has presumably a more stable bonding environment at the interface due to S-termination that has been interpreted to diminish e.g. dangling bonds. Both of these effects can suppress out-diffusion of In.

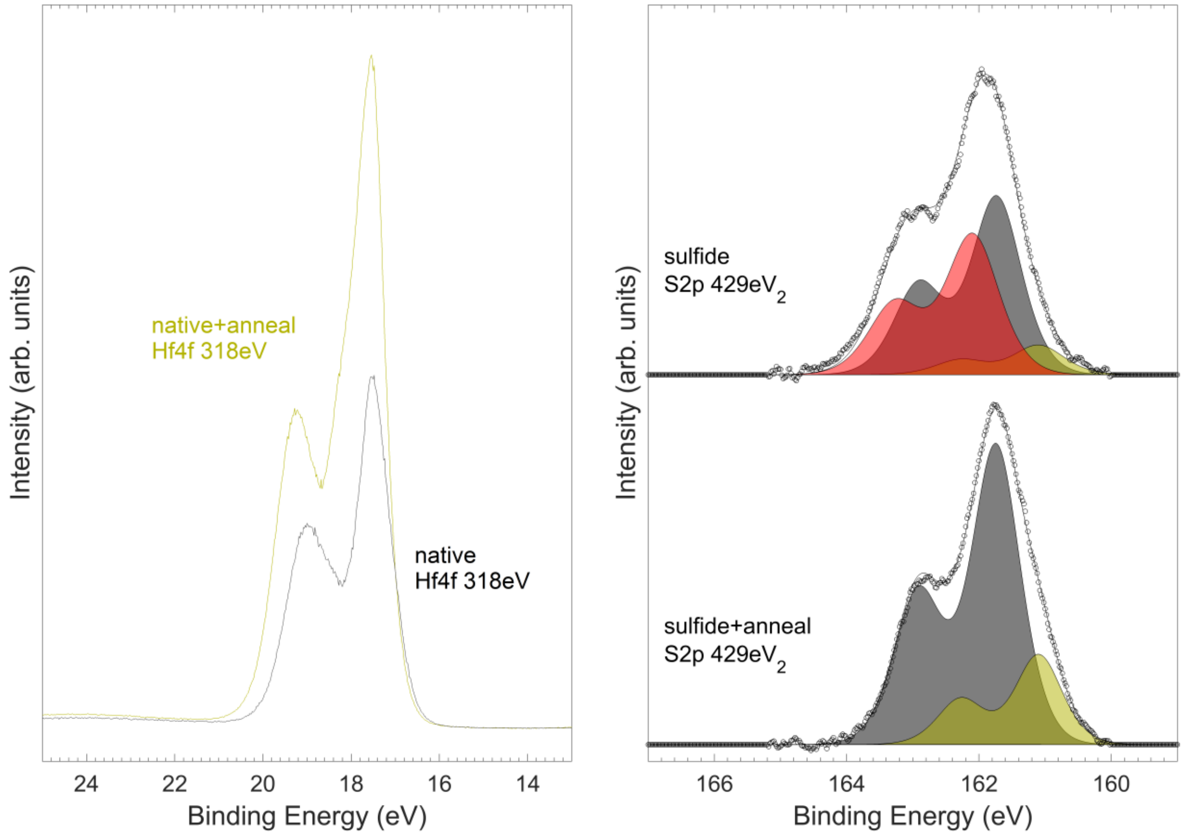

Fig. S1. *Hf 4f (and overlapping In 4d, without fitting) and S 2p spectra. S 2p has been fitted with three peak doublets, highest BE component corresponding to excess O-bonds and lower BE components to compounds with higher coordination to Hf, In, or P (sulfide compounds).*

[44] J. F. Moulder, W. F. Stickle, P. E. Sobol, K. D. Bomben, *Handbook of X-ray photoelectron spectroscopy*, Vol. 40 (Perkin Elmer Eden Prairie, MN, 1992)
